# Association between Dietary Patterns and Frailty Prevalence in Shanghai Suburban Elders: A Cross-Sectional Study

**DOI:** 10.3390/ijerph182010852

**Published:** 2021-10-15

**Authors:** Yingchuan Wang, Yue Huang, Han Wu, Gengsheng He, Shuguang Li, Bo Chen

**Affiliations:** 1 Department of Nutrition and Food Hygiene, Key Laboratory of Public Health Safety of Ministry of Education, School of Public Health, Fudan University, Shanghai 200032, China; 17211020138@fudan.edu.cn (Y.W.); 17211020098@fudan.edu.cn (H.W.); gshe@shmu.edu.cn (G.H.); leeshuguang@fudan.edu.cn (S.L.); 2 Department of Food Science and Nutrition, Shanghai Business School, Shanghai 200235, China

**Keywords:** dietary pattern, elderly, frailty, DASH, Chinese

## Abstract

Objective: To investigate the association between dietary patterns with frailty phenotypes in an elderly Chinese population. Methods: A cross-sectional study was performed in 780 Shanghai suburban elders aged 65–74 in 2019. Dietary data were collected using a food frequency questionnaire. Adherence to a priori dietary patterns, including the Chinese Healthy Eating Index (CHEI), Dietary Approaches to Stop Hypertension (DASH) diet and Mediterranean Diet (MD) were calculated. Three a posteriori dietary patterns were identified by factor analysis, namely, “protein-rich”; “vegetables”; and “sugar, oil, and condiments”. Frailty was defined using the Fried frailty phenotype scale. Ordinal multiple logistic regression was applied to examine the associations between dietary patterns and frailty prevalence. Results: The prevalences of pre-frailty and frailty were 47.69% and 3.85%, respectively. Participants with a higher DASH score had a lower frailty prevalence in the sex- and age-adjusted models of the 780 subjects (OR = 0.97 (95% CI: 0.94–0.99), *p* < 0.05). The association slightly strengthened in the multivariate adjusted model of the 555 subjects after excluding the participants with chronic diseases may influence frailty (OR = 0.96 (95% CI: 0.92–1.00), *p* < 0.05). High “protein-rich” dietary pattern scores were negatively associated with frailty prevalence in the multivariate adjusted model of the 780 subjects (OR = 0.82 (95% CI: 0.69–0.98), *p* < 0.05). The association attenuated in the sex- and age-adjusted model of the 555 subjects (OR = 0.84 (95% CI: 0.69–1.00, *p* = 0.056). Conclusion: A better diet quality as characterized by DASH and “protein-rich” was associated with a reduced prevalence of frailty in Shanghai suburban elders. The correlation of CHEI, MD or a posteriori dietary patterns with the development of frailty in Chinese older people remains to be explored.

## 1. Introduction

Frailty is a complex age-related clinical condition characterized by a decline in physiological capacity across several organ systems, with a resultant increased susceptibility to stressors [1]. The rapid expansion of the ageing population has brought a concomitant rise in the number of older adults with frailty, which in turn places an increased pressure on health-care systems worldwide [2,3]. Accordingly, a better understanding of the determinants of frailty is needed to support evidence-based prevention interventions of improving both health status and productive longevity of older adults. Certain modifiable environmental factors such as lifestyle, physical activity level, and diet can affect the degenerative aging process [4]. Among these factors, theoretically, nutrition is a relative importance one closely related to the frailty syndrome, since all frailty criteria are more or less affected by poor eating habits. For example, chronic undernutrition and insufficient protein and energy intake lead to weight loss and sarcopenia, which may, in turn, cause low muscle strength and feeling of exhaustion. On the other hand, frailty itself may have a negative effect on eating and, thus, on the nutritional status [5,6].

Research on dietary determinants of frailty is relatively recent. To date, studies have supported that low protein, macronutrient, and micronutrient intake is linked to frailty [5,6,7]. However, such association between single nutrient and frailty fails to consider the interactions between nutrients; investigations in recent years were therefore shifted toward evaluating the effects of dietary patterns [8]. Dietary patterns are derived by two approaches: an a priori approach, which scores an individual’s adherence to the recommended dietary guidelines or predefined dietary pattern; and an a posteriori approach, which uses statistical exploratory methods to identify dietary patterns on the basis of the individual’s dietary intake [9,10].

So far, the findings were interested and rather inconclusive, which may have resulted from different patterns of dietary behaviors in different populations. For instance, several studies in Europe and the United States revealed that better adherence to the Mediterranean diet, characterized as abundance in fruits, vegetables, whole grains, legumes, and olive oil, was associated with lower incident frailty risk in the elderly [11,12]. By contrast, a two-year cohort in Hong Kong found that the Mediterranean diet failed to prevent the risk of frailty [13]. In addition, the Dietary Approaches to Stop Hypertension (DASH) score was associated with lower chronic disease and mortality risk [14,15,16,17,18], while its relationship with frailty has not been established. Moreover, other a priori and a posteriori dietary patterns have also found inconsistent results [19,20,21,22,23].

More notably, some of the diet quality scores are dependent on the sample data, and comparison of the results across populations is very difficult. Thus, it would be meaningful to understand the relationship between the diet quality scores and frailty in the Chinese population, as well as the impact of other a posteriori dietary patterns on frailty prevalence. The present study aimed to examine the relationship of a priori and a posteriori diet patterns with frailty prevalence in Chinese community-dwelling older people in Shanghai.

## 2. Materials and Methods

### 2.1. Study Design and Participants

Participants were recruited from the Shanghai Suburban Adult Cohort and Biobank (SSACB) survey. SSACB is a population-based, large-sized natural cohort study that uses a representative sample living in a suburban area with rapid urbanization. Details of this cohort have been described previously [24,25]. In brief, the target population were native Chinese residents (age 20–74 years old), and a stratified clustered sampling design was used. Residents were randomly selected by multi-stage sampling. The cohort started to complete a baseline survey of about 40,000 people in representative streets in Songjiang district in 2016, with Zhongshan Street as one of the representative streets, which completed a baseline survey of 7098 people from April to September 2017. Among the above baseline population from Zhongshan Street, a sub-sample of 896 participants aged 65 years or above was randomly selected and invited to participate in the frailty survey from April to May 2019. The participants enrolled fulfilled the following inclusion criteria: (1) able to walk or take public transport to the study site; (2) able to communicate normally, without serious cognitive and psychiatric disorders; (3) willing to cooperate with the investigators, as well as the following exclusion criteria: (1) failed to complete the physical assessment test; (2) with serious diseases or unconsciousness. This study was conducted in compliance with the Declaration of Helsinki and was approved by the Institutional Review Board of Fudan University (IRB#2016-04-0586). All participants gave written informed consent. A face-to-face questionnaire and a physical examination were administered at participants’ homes and temporary health examination stations to gather the corresponding information on dietary pattern and frailty. After excluding 116 participants with incomplete data of demographic, characteristic, physical examinational information, or frailty survey information and with unreasonable energy intake (male: <800 kcal or >4000 kcal; female: <500 kcal or >3500 kcal), the sample size for the final analysis was 780.

### 2.2. Questionnaire and Anthropometric Measurements

A standardized interview was conducted to capture information on socio-demographic characteristics, lifestyle and previous health status during the baseline survey. Socio-demographic information such as age, sex, education, marital status, type of occupation prior to retirement, and annual per capita household income was investigated. Lifestyle variables included smoking and alcohol intake, housekeeping, and sedentary behaviors. Health status variables included self-reported history of diabetes, hypertension, angina, stroke, arthritis, chronic lung disease, asthma, myocardial infarction, congestive heart failure, kidney disease, and cancer. Body weight was measured by trained health worker to the nearest 0.1 kg with the participant standing without shoes and wearing light clothing, and height was measured without shoes to the nearest 0.1 cm using an electronic height and weight scales. Body mass index (BMI) was calculated as body weight in kg/(height in m)^2^. 

### 2.3. Frailty Assessment

The five-item scales based on Fried frailty phenotype were used to assess frailty status [26]. A score of 1 is assigned to each of the five components: weight loss (unintentional weight loss of >5% or 4.5kg in the previous year, or BMI < 18.5), exhaustion (a positive response to the 2 items “I felt that everything I did was an effort”, and “I could not get going.” from the Centres for Epidemiologic Studies Depression Scale (CES-D: 10 items) during the past 4 weeks), low physical activity (<383 Kcals per week in men, <270 Kcals per week in women, assessed using a Physical Activities Questionnaire to ascertain the time spent on selected physical activities), slow walking speed (≥5 seconds to walk 4 m), and weak grip strength (men ≤ 29–32 kg, women ≤ 17–21 kg, stratified by BMI quartiles for maximum strength in the dominant hand, measured with a dynamometer). If a component was present, 1 point was allocated for that component; if a component was absent, the score was 0. Frailty scores range from 0 to 5 and represent frail (3 to 5), pre-frail (1 to 2), and robust (0) health status.

### 2.4. Dietary Assessment

Dietary intake was assessed using a validated, semi-quantitative food frequency questionnaire (FFQ) adapted from an existing FFQ used in the Shanghai Diet and Health Study since 2010 [27], which containing 29 food groups comprised by similar food items (e.g., rice and rice products, wheat and wheat products, dark vegetables, fruits, dairy and dairy products, and so on), representing approximately 95% of the most commonly consumed foods in Shanghai. Each food group forms one question. In the present study, all participants were asked to recall the consumption frequency of each food group in the previous 12 months (never, less than 1 time/month, 1–3 times/ month, 1–3 times/week, 4–6 times/week, 1 time/d, 2 times/d, 3 times/d) and the estimated portion size. Portion size was quantified using a catalogue of pictures of individual food portions. Data from the FFQ were then converted to daily amount of consumption of major food groups. The amounts of oil, sugar, salt, soy sauce, and other condiments reported for the entire family were divided by the number of family members and then divided by the proportion of meals consumed at home and converted to daily intake. Mean daily nutrient intake was calculated by multiplying the consumption of each food by its nutrient content, using food tables derived from the Chinese Medical Sciences Institute [28].

The reliability of the FFQ was assessed by comparing two-times (first and second) FFQ at an interval of 12 months in 152 participants randomly selected from baseline samples of SSACB at Zhongshan Street. Similarly, the validity was assessed by comparing the second FFQ with 3-day 24-hour dietary recalls in 165 participants. Both reliability and validity show satisfied coefficients in energy-adjusted correlation analyses of both nutrients (reliability: 0.39–0.60; validity: 0.12–0.42) and food groups (reliability: 0.36–0.54; validity: 0.20–0.41).

### 2.5. A Priori Dietary Pattern Scores

The adherence to 3 diet quality scores of the Chinese Healthy Eating Index (CHEI), Dietary Approaches to Stop Hypertension (DASH) diet and Mediterranean Diet (MD) were calculated. The 3 scores range from 0 to 100 (CHEI), 8 to 40 (DASH), and 0 to 9 (MD), with higher scores indicating better adherence to the dietary pattern (Supplementary Materials, Table S1).

The CHEI is an instrument in China with good validity and reliability to evaluate the overall diet quality in accordance with the updated Dietary Guidelines for Chinese (DGC-2016) [29,30]. In brief, the CHEI comprises 17 components, 12 of which evaluate the adequacy of a diet, including total grains, whole grains and mixed beans, tubers, total vegetables, dark vegetables, fruits, dairy, soybeans, fish and seafood, poultry, eggs, seeds, and nuts. The other 5 components assess the limitation of a diet, including red meat, cooking oils, sodium, added sugars, and alcohol. Higher intakes of adequacy components will receive higher scores, whereas higher intakes of limitation components will receive lower scores. All of the 17 components are summarized to obtain a total score, which ranges from 0 to 100 with a higher score indicating a better diet quality.

The DASH and MD scores are two widely used dietary indices to measure the diet quality and association with diseases. The DASH diet was originally designed to promote blood pressure reduction. In the DASH score, 1 to 5 points were assigned based on quintiles of intake in servings per day of fruits, vegetables, nuts and legumes, low-fat dairy products, and whole grains. Scoring was inverse for sodium, sugar-sweetened beverages, and red and processed meat, with more points for lower consumption. The total score ranged from 8 to 40 (highest adherence) points [31]. The MD score was calculated to assess the adherence to the Mediterranean diet [32]. Essentially, adherence is represented by a scale where a value of 1 was assigned to consumption of food groups considered beneficial to health at or above the sex-specific median (vegetables, legumes, fruits and nuts, cereal, fish, and monosaturated to saturated lipids ratio) and below the median for food groups presumed to be detrimental to health (meat, poultry, and dairy products). For ethanol consumption, a value of 1 was assigned to men who consumed between 10 and 50 g per day and to women who consumed between 5 and 25 g per day. Therefore, a higher MD score corresponds to a higher adherence to the Mediterranean diet, with a final range from 0 to 9 points.

### 2.6. A Posteriori Dietary Pattern Scores

To identify dietary patterns, individual food items from the FFQ were first aggregated into 27 food groups according to the similarity of food type and nutrient composition (Supplementary Materials, Table S2). Factor analysis was performed with varimax rotation using the 27 food groups. Factors were retained based on an eigenvalues greater than 1.0, a scree plot, and the interpretability. The dietary pattern was named according to the larger factor load on each factor (FL > 0.2). The factor scores for each pattern were then calculated for each participant through summing intakes of food items weighted by their factor loadings. A higher score represented greater conformity with the derived pattern. Three dietary patterns were identified in the present study, namely, “protein-rich”; “vegetables”; and “sugar, oil, and condiments”, which explained 8.81%, 6.53%, and 6.22% of the variation, respectively (Table 1). 

### 2.7. Statistical Analysis

Descriptive statistics were conducted to ascertain the demographic characteristics of the sample (presented as means and SD, or as numbers and percentages for categorical data). Data were checked for normality using descriptive analysis. Independent student’s *t* test, one-way ANOVA and chi square test were used to examine the group differences of the participants. Ordinal multiple logistic regression models were used to analyze the association between each diet pattern score and the prevalence of pre-frailty and frailty and further adjusted for covariates that may affect frailty. The first model was the unadjusted model, and the second model was adjusted for sex and age (continuous). The third model was further adjusted for BMI (continuous), daily energy intake (continuous), household income (categorical), marital status (categorical), education level (categorical), housekeeping behaviors (yes vs. no), current smoker status (yes vs. no), current alcohol status (yes vs. no). 

Then, after excluding those participants with chronic diseases may influence frailty, further analysis using the same ordinal multiple logistic regression models was performed to analyze the association. A 2-sided *p* < 0.05 was considered to be statistically significant. Statistical analyses were performed using SPSS v25.0 (SPSS Inc., Chicago, IL, USA). 

## 3. Results

### 3.1. General Information of the Participants

A total of 780 elderly residents aged 65–74 years were included in this study, with a mean age of 66.85 years (SD = 2.64), mean BMI of 25.35 kg/m^2^ (SD = 3.29) and mean daily energy intake of 1408.39 kcal (SD = 579.45). There were 445 females (57.05%) and 325 males (42.95%) in the investigated population. The majority of participants were 65–69 years old (60.51%), married (86.15%), had achieved an educational level of primary school or below (78.59%), and had an annual per capita household income of ￥10,000 to ￥30,000 (63.46%). The prevalence of smoking and drinking was 18.97% and 16.54%, respectively. Most of them were doing housework every day (86.15%). Only 12.05% of participants were sedentary for more than 6 h a day. Many of them had several kinds of self-reported diseases. 

The percentages of robustness, pre-frailty and frailty were 48.46%, 47.69%, and 3.85%, respectively. The frailty distribution differences in groups of sex, age, education level, marital status, household income, current smoker status, current alcohol status, housekeeping behaviors, self-reported diseases of diabetes, asthma, and stroke were significant (*p* < 0.05), while the difference between the groups of being sedentary behaviors or not was not observed. Participant characteristics and distribution of frail status were shown in Table 2.

### 3.2. Dietary Pattern Scores of the Participants 

**Figure 1:**
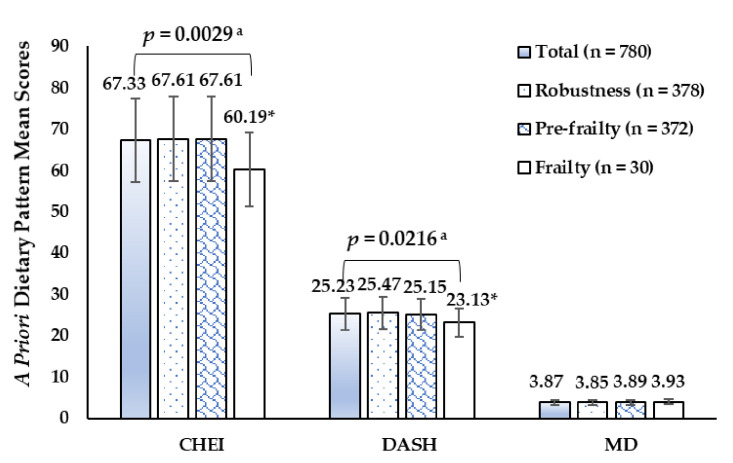
A priori dietary pattern mean sores of participants by frail status. ^a^ *p*-value between robust, pre-frail and frail participants by one-way *ANOVA*, Bonferroni post hoc test. * *p* < 0.05, compared with the means in Robust groups.

As shown in Figure 1, a priori diet quality scores on CHEI, DASH, and MD were 67.33 (SD 11.69), 25.23 (SD 4.50), and 3.87 (SD 1.43) in the present investigation. The CHEI and DASH scores in the frail group were lower than those in the robust group (60.19 vs. 67.61, 23.13 vs. 25.47, respectively) (*p* < 0.05). There were no statistically significant differences in MD scores among study populations with different frail status.

**Table 2:** Characteristics of participants and distribution of frailty (*n* = 780).

Characteristics ^a^	Total	Robustness	Pre-Frailty	Frailty	*p* Value ^b^
**Age (Years)**	68.85 (2.64)	68.57 (2.60)	69.14 (2.66)	68.87 (2.66)	**0.011**
**BMI (kg/m^2^)**	25.35 (3.29)	25.04 (2.98)	25.63 (3.53)	25.80 (3.75)	**0.041**
**Energy intake (kcal/day)**	1408.39 (579.45)	1438.06 (586.89)	1388.88 (579.01)	1276.43 (467.06)	0.227
**Total, *N* (%)**	780 (100)	378 (48.46)	372 (47.69)	30 (3.85)	
**Sex**					
Male	335 (42.95)	191 (57.01)	136 (40.60)	8 (2.39)	**<0.001**
Female	445 (57.05)	187 (42.02)	236 (53.03)	22 (4.94)	
**Age**					
65–69 years	472 (60.51)	247 (52.33)	208 (44.07)	17 (3.60)	**0.010**
70–74 years	308 (39.49)	131 (42.53)	164 (53.25)	13 (4.22)	
**Education Level**					
Primary school or below	613 (78.59)	279 (45.51)	307 (50.08)	27 (4.40)	**0.004**
Secondary school or above	167 (21.41)	99(59.28)	65 (38.92)	3 (1.80)	
**Marital status**					
Married	672 (86.15)	339 (50.45)	307 (45.68)	26 (3.87)	**0.018**
Others ^c^	108 (13.85)	39 (36.11)	65 (60.19)	4 (3.70)	
**Annual per capita household income**					
<￥10,000	58(7.44)	19 (32.76)	35 (60.34)	4 (6.90)	**<0.001**
￥10,000~30,000	495 (63.46)	223 (45.05)	252 (50.91)	20 (4.04)	
≥￥30,000	227 (29.10)	136 (59.91)	85 (37.44)	6 (2.64)	
**Behavioral variables**					
Current smoker ^d^	111 (14.23)	63 (56.76)	42 (37.84)	6 (5.41)	**0.006**
Not current smoker	669 (85.77)	315 (47.09)	330 (42.31)	24 (0.04)	
Current alcohol use ^e^	50 (6.41)	17 (34.00)	29 (58.00)	4 (8.00)	**<0.001**
Not current alcohol user	730 (93.59)	361 (49.45)	343 (46.99)	26 (3.56)	
Doing housework everyday	672 (86.15)	329 (48.96)	324 (48.21)	19 (2.83)	**0.001**
Not doing housework everyday	108 (13.85)	49 (45.37)	48 (44.44)	11 (10.19)	
Being sedentary > 6 h/day	94 (12.05)	45 (47.87)	43 (45.74)	6 (6.38)	0.390
Not being sedentary > 6 h/day	686 (87.95)	333 (48.54)	329 (47.96)	24 (3.50)	
**Self-reported diseases**					
Hypertension	405 (51.92)	198 (48.89)	189 (46.67)	18 (4.44)	0.606
Without hypertension	375 (48.08)	180 (48.00)	183 (48.80)	12 (3.20)	
**Diabetes**	129 (16.54)	52 (40.31)	62 (48.06)	15 (11.63)	**<0.001**
Without diabetes	651 (93.46)	326 (50.08)	310 (47.62)	15 (2.30)	
Chronic lung disease	50 (6.41)	20 (40.00)	25 (50.00)	5 (10.00)	0.067
Without chronic lung disease	730 (93.59)	358 (49.04)	347 (47.53)	25 (3.42)	
Myocardial infarction	17 (2.18)	6 (35.29)	10 (58.82)	1 (5.88)	0.465
Without myocardial infarction	763 (97.92)	372 (48.75)	362 (47.44)	29 (3.80)	
Angina	33 (4.23)	14 (42.42)	17 (51.52)	2 (6.06)	0.669
Without angina	747 (95.77)	364 (48.73)	355 (47.52)	28 (3.75)	
**Asthma**	26 (3.33)	8 (30.77)	13 (50.00)	5 (19.23)	**<0.001**
Without asthma	754 (96.67)	370 (49.07)	359 (47.61)	25 (3.32)	
Arthritis	286 (36.67)	132 (46.15)	139 (48.60)	15 (5.24)	0.238
Without arthritis	494 (63.33)	246 (49.80)	233 (47.17)	15 (3.04)	
**Stroke**	102 (13.08)	37 (36.27)	56 (54.90)	9 (8.82)	**0.002**
Without stroke	678 (86.92)	341 (50.29)	316 (46.61)	21 (3.10)	
Kidney disease	164 (21.03)	80 (48.78)	78 (47.56)	6 (3.66)	0.988
Without kidney disease	616 (79.97)	298 (48.38)	294 (47.73)	24 (3.90)	
Cancer	27 (3.46)	8 (29.63)	17 (62.96)	2 (7.41)	0.114
Without cancer	753 (96.54)	370 (49.14)	355 (47.14)	28 (3.72)	

^a^ Categorical variables are presented as frequency (percentages) based on chi square test or Fisher exact test. Continuous variables are presented as mean (standard deviation). ^b^ *p*-value between robust, pre-frail, and frail participants by one-way ANOVA for continuous variables and chi square test or Fisher exact test for categorical variables. ^c^ Widowed, Separated, divorced, single, or never married. ^d^ Smoking at least once a day for at least six months. ^e^ Drinking alcohol at least three times a week for at least six months. *p* values < 0.05 are shown in bold.

**Figure 2:**
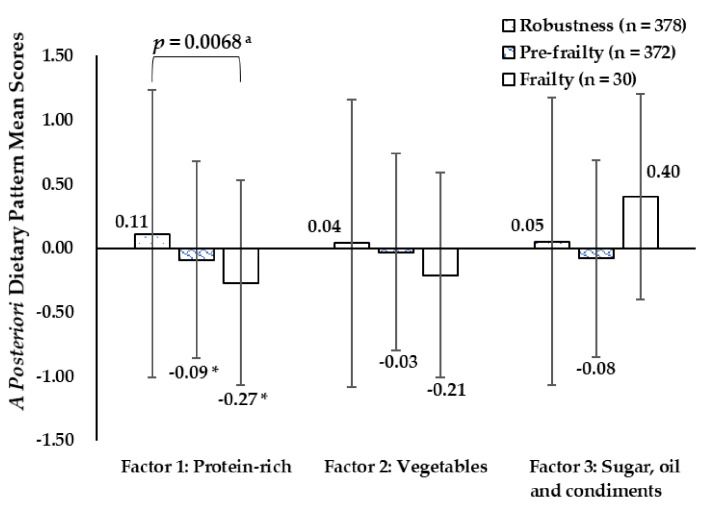
A posteriori dietary pattern mean sores of participants by frail status. ^a^ *p*-value between robust, pre-frail, and frail participants by one-way *ANOVA*, Bonferroni post hoc test. * *p* < 0.05, compared with the means in Robust groups.

The a posteriori dietary pattern mean scores of participants by frail status were presented in Figure 2. The “protein-rich” dietary pattern scores were significantly different in the robust, pre-frail, and frail groups (0.11 vs −0.09 vs −0.27) (*p* < 0.05). There were no statistically significant differences in “vegetables” and “sugar, oil, and condiments” dietary pattern scores among study populations with different frail status.

Some sex and age differences in dietary pattern scores were observed (Table 3). Females got higher CHEI, DASH, and MD scores than males (*p* < 0.05), while the mean score of the “protein-rich” dietary pattern was much lower in females than in males (*p* < 0.01). Participants aged 65–69 scored higher on CHEI, DASH, and “protein-rich” diet pattern than those aged 70–74 (*p* < 0.05).

### 3.3. Associations between Each Dietary Pattern Score and Frailty Prevalence

Participants with a higher DASH score had a lower frailty prevalence in the sex- and age-adjusted models; the OR was 0.97 (95% CI: 0.94–0.99) (*p* < 0.05). Further analysis after excluding those with chronic diseases may influence frailty showed that the association slightly strengthened in the 555 subjects without diabetes, stroke, or asthma. The OR was 0.96 (95% CI: 0.92–1.00) (*p* < 0.05) in the model adjusted for demographic and lifestyle factors including sex, age, BMI, energy intake, educational level, marital status, household income, smoking status, alcohol use, and doing housework, indicating that every one-SD increase in DASH scores reduced the frailty prevalence by about 4% (Table 4).

There were significant correlations between high “protein-rich” dietary pattern scores and frailty prevalence in the unadjusted and adjusted models in the 780 participants. The OR was 0.82 (95% CI: 0.69–0.98) (*p* < 0.05) in the model adjusted for socio-demographic and lifestyle factors, which showed that high “protein-rich” dietary pattern scores were negatively associated with frailty prevalence after controlling for covariates including sex, age, BMI, energy intake, educational level, marital status, household income, smoking status, alcohol use, and doing housework. However, the associations had a slight attenuation after excluding the participants with chronic diseases which may influence frailty including diabetes, stroke, and asthma. The OR was 0.84 (95% CI: 0.69–1.00) (*p* < 0.05) in the sex- and age-adjusted model. When the model was further adjusted for other socio-demographic and lifestyle factors, the associations were not significant. 

There was no association of CHEI; MD; “vegetables”; or “sugar, oil, and condiments” dietary pattern with frailty.

## 4. Discussion

Frailty is a biological syndrome resulting from multisystem impairments associated with aging, and prefrailty is an intermediate state between frailty and robustness, with a high risk of progressing to frailty [33]. Currently, no accepted reference standard exists to identify frailty, and extensive international efforts are underway to identify the means of optimal measurement. The most frequently used approach to define frailty is the Fried physical phenotype model [26], which we used in the present study. The prevalences of pre-frailty and frailty were 47.69% and 3.85% in the community-dwelling older people aged 65–74 in the Shanghai suburban area, which showed that more than half of the participants had frailty-related symptoms. This alarmingly high prevalence estimated in our study revealed an urgent need for preventative intervention in frailty among Chinese community-dwelling older people.

We found that there were more prefrail and frail individuals in female and people aged 70–74, and these frailty distribution differences between sex and age groups may be related to many factors such as health status and dietary and physical activity behaviors. We also found that some sex and age differences in dietary pattern scores were significant. Specifically, the “protein-rich” dietary pattern scores were much lower in female and people aged 70–74, which may be part of the potential factors to explain the sex and age differences of frailty distribution.

Optimal nutrition is important for healthy ageing and is presumably an important factor that can prevent the development of frailty [6]. Diet quality indices or scores, the measuring instrument of diet quality, are employed to summarize dietary intake into a single numeric variable, which addresses the limitations in the assessment of diet-disease relationships based only on a single food or a single nutrient [34]. In this cross-sectional study, three a priori dietary pattern sores of CHEI, DASH, and MD were assessed. The three indices have common characteristics, they all award more points for higher intake of fruits, vegetables, nuts, and fiber and lower intake of red meat. The commonalities in these indices may help to reveal key dietary components to further investigate and unravel the relationship between diet and frailty. This is the first study to our knowledge to investigate the association of the CHEI and DASH scores and frailty prevalence in a Chinese population. Results showed that the DASH scores in the frail group were lower than those in the robust group. 

The DASH diet emphasizes lower intakes of foods related to higher risk of hypertension (sodium, red and processed meats, and sweetened beverages) and higher intake of foods related to lower risk of hypertension (fruit, vegetables, nuts, legumes, low-fat dairy, and whole grains) [18], which was originally developed to treat hypertension without medication and then proved to be associated with lower risk of many chronic diseases such as diabetes, metabolic syndrome, kidney, and cardiovascular diseases [14,15,16,17,18]. However, studies on the association of DASH diet with frailty are relatively limited currently. A recent study reported that after adjusting for potential confounders, adherence to a healthy diet as defined by the DASH scores was associated with reduced risk of frailty in 71,941 older women in the US [35]. In our study, results showed that higher DASH scores were associated with lower frailty prevalence among older adults, suggesting that a better diet quality measured using DASH dietary indices may help to reduce the frailty prevalence in older people. This finding was consistent with that of a recent cross-sectional analysis among 9861 male physicians in the US, which showed an inverse dose-response relationship of DASH quintiles with pre-frailty and frailty [36]. There are several possible mechanisms for this phenomenon, for instance, adherence to a healthy diet was associated with a reduced risk of chronic diseases including cardiovascular disease that shares a bidirectional relationship with frailty [37]; oxidative stress may be a mechanism contributing to frailty, and diets high in fruit, vegetables, legumes, and whole grains were associated with lower inflammatory markers and higher levels of antioxidant micronutrients, which may reduce oxidative stress [38]; insulin resistance, favored by added sugars and sodium intake, may also produce macro- and microvascular complications and promotes cognitive impairment in the elderly, which also contribute to frailty [39]. 

The CHEI evaluates the overall diet quality in accordance with the Dietary Guidelines for Chinese, representing a balanced diet in terms of energy and nutrient intakes as well as various food groups consumption [29,30], which was expected to play a beneficial role in frailty prevention. However, there were no significant correlations between the CHEI scores and frailty prevalence in the further ordinal multiple logistic regression analysis; this may be due to the same even CHEI scores in the pre-frail and robust groups and could be partly explained by the observational and cross-sectional design of this study. Most of the participants were very familiar with the Dietary Guidelines for Chinese (DGC)–2016, and the individuals physically weaker were more likely to change their previous diet patterns according to DGC–2016 in daily life, which may lead to a reverse cause-and-effect relationship in cross-sectional studies. The relationship between the CHEI scores and frailty deserve further investigation in future longitudinal or interventional studies.

In general, a Mediterranean-style diet is associated with a high intake of healthy food (vegetables, fruit, nuts, legumes, whole grains, and fish), a healthy monounsaturated fat-to-saturated fat ratio (olive oil as the principal source of fat), and a low to moderate intake of red and processed meat and alcohol (wine) [12]. Several previous studies demonstrated the association between a higher adherence to MD with a lower risk of frailty in old people [40,41,42]. Nevertheless, the benefits may be more obvious among elders from western countries, no significant associations were found among elders from Asia [13,22]. In the present study, there was no association of MD with frailty prevalence. The difference in eating habits between western and eastern countries may contribute to this phenomenon. Although the Mediterranean diet and the dietary pattern recommended by Dietary Guidelines for Chinese share some similar characteristics, such as high fruit and vegetable consumption and low meat consumption, there are substantial differences [12]. In particular, the consumption of olive oil and grape wine typical of the Mediterranean diet was much less common in our studied population. 

Three a posteriori dietary patterns were identified in the current study, and only a significant correlation between “protein-rich” dietary pattern scores with frailty prevalence was observed, illustrating that the dietary pattern with higher intake of protein-rich foods such as soybean and soybean products, meat and poultry, and fish and shellfish was inversely associated with frailty, which was in line with a recent research in Japan [43]. The role of protein in offsetting frailty may be attributed to its contribution to muscle and bone health [44]. Older adults need more proteins to preserve muscle mass compared to younger people [45]. Substantial evidence suggests that protein intake has a key role in stimulating muscle protein synthesis and, consequently, in the preservation of muscle mass and physical performance [46,47]. Although emerging observational studies had reported an inverse association between dietary protein-related parameters and frailty-related parameters in older adults worldwide in recent years [48,49,50], the results were not unanimous among studies [43,51,52,53]. Different protein-related parameters, such as protein intake, quality and distribution across meals, and different instruments for assessing frailty may capture different aspects of the association between frailty and protein [46,54], which deserves further in-depth study.

Frailty is a potentially reversible and transitional state in the dynamic progression from robustness to functional decline [55]. Therefore, interventions for frailty could be practical, and may have profound benefits in improving the life quality of elderly people. Future intervention is expected to focus on the contribution of nutrition, especially for the “protein-rich” and DASH diet, to offset and reverse the frail condition or delay its onset and progression.

Several limitations need to be acknowledged in this study. First, our participants were volunteers who in good physical condition and living in a suburban area; therefore, the extrapolation of results to the general population should be made with caution. In addition, the a posteriori dietary pattern approach is dependent on the sample information; the pattern derived is therefore highly specific to the diet of the studied population. The results or patterns generated from this study may not be generalized to other target populations. Second, the dietary indices and frailty measures relied on self-reported data; factors such as age-related decline in cognitive function may have contributed to some measurement error. Especially, the FFQs are based on the recall of each participant and therefore may lead to recall bias. Third, although we did adjust for many potential covariates, residual and unmeasured confounding cannot be completely ruled out. Finally, given the cross-sectional design of this study, the observed relationships cannot be interpreted as causal and remain to be confirmed in prospective studies, especially when subjects with frailty may change diets leading to reverse causality. The studied population in our data had relatively consistent diets over 12 months as assessed by satisfied reliability of the FFQ. Literature also reported the consistent habits of diets in Chinese elders [56,57]. The functional decline associated with frailty is gradual and incessant. Compared with other diseases, frailty has the slowest rate of functional decline observable in daily life [1,58]. It may be reasonable to speculate that elders with pre-frailty have consistent habits of diets. In such situation, the causality from diets to frailty may be more reasonable than the reverse one. Future studies using longitudinal data and intervention trials are expected to better explain or prove such causality.

## 5. Conclusions

In conclusion, a better diet quality as characterized by DASH and “protein-rich” was associated with a reduced prevalence of frailty in Shanghai suburban elders. Further studies are needed to confirm this association and investigate whether related dietary interventions can reduce frailty in older adults. The correlation of CHEI, MD, or a posteriori dietary patterns with the development of frailty in Chinese older people remains to be explored.

## Figures and Tables

**Table 1:** Factor loadings to determine the association between food groups and factors representing dietary patterns ^a^.

Food Items	Dietary Patterns ^b^		
	Factor 1: “Protein-Rich”	Factor 2: “Vegetables”	Factor 3: “Sugar, Oil, and Condiments”
Soybean and soybean products	**0.66**	0.26	0.10
Red meat	**0.58**	0.04	−0.04
Poultry	**0.39**	0.17	−0.08
Freshwater fish	**0.63**	0.15	0.04
Ocean fish	**0.44**	0.40	−0.06
Shellfish, Shrimp, and Crab	**0.74**	0.05	−0.07
Dark vegetables	0.01	**0.88**	0.02
Light colored vegetables	0.38	**0.74**	0.04
Added sugars	−0.03	0.02	**0.58**
Cooking oil	0.06	−0.01	**0.75**
Condiments	−0.05	0.05	**0.79**
Fruits	0.19	−0.02	0.17
Juice	0.00	−0.02	−0.02
Eggs	0.04	0.17	−0.03
Dairy and dairy products	0.02	0.19	0.09
Processed meat	0.07	0.01	0.00
Animal innards	0.11	−0.03	−0.01
Rice and rice products	−0.05	0.22	−0.01
Wheat and wheat products	0.07	0.05	−0.02
Fried dough foods and potato chips	0.01	−0.04	0.02
Whole Grains and Mixed Beans	−0.06	0.14	−0.09
Tubers	0.12	0.00	−0.01
Nuts and Seeds	0.08	−0.05	0.07
Sweets and desserts	0.00	0.01	0.08
Cakes, cookies, pies, and biscuits	0.02	−0.02	−0.02
Beverages	−0.02	0.02	0.05
Alcoholic beverages	0.08	−0.08	0.23

^a^ with promax rotation, the factor loading scores are identical to the correlation coefficients. Factor loadings with absolute value > 0.20 are shown in bold. For food group loads more than one dietary pattern, only the highest absolute value of loading is bolded. ^b^ Explained variance: “Protein-rich” dietary pattern, 8.81%; “vegetables” dietary pattern, 6.53%; and “sugar, oil, and condiments” dietary pattern, 6.22%.

**Table 3:** Dietary pattern scores of participants by sex and age groups (*n* = 780).

Dietary Pattern Score, Mean (SD)	Sex			Age		
	Male (*n* = 335)	Female (*n* = 445)	*p* value ^a^	65–59 (*n* = 472)	70–74 (*n* = 308)	*p* Value ^a^
CHEI	66.10 (11.58)	68.25 (11.70)	**0.011**	68.18 (11.55)	66.02 (11.80)	**0.012**
DASH	24.79 (4.57)	25.55 (4.43)	**0.020**	25.52 (4.56)	24.77 (4.39)	**0.023**
MD	3.54 (1.37)	4.13 (1.42)	**<0.001**	3.94 (1.45)	3.77 (1.38)	0.108
Factor 1: Protein-rich	0.12 (1.16)	−0.09 (0.85)	**0.005**	0.09 (1.12)	−0.13 (0.75)	**0.003**
Factor 2: Vegetables	0.02 (0.70)	−0.02 (1.18)	0.605	0.02 (1.13)	−0.02 (0.76)	0.686
Factor 3: Sugar, oil and condiments	0.07 (1.14)	−0.05 (0.88)	0.104	−0.04 (1.07)	0.06 (0.88)	0.160

CHEI: Chinese Healthy Eating Index; DASH: Dietary Approaches to Stop Hypertension; MD: Mediterranean Diet; ^a^ *p*-value between sex and age groups by independent samples *t*-tests; the *p* values < 0.05 level are shown in bold.

**Table 4:** Crude and adjusted associations between each dietary pattern score and frailty prevalence.

Dietary Pattern Score	Model 1			Model 2 ^a^			Model 3 ^b^		
	OR	95%CI	*p* Value	OR	95%CI	*p* Value	OR	95%CI	*p* Value
**All 780 subjects**									
DASH	0.97	0.94–1.00	**0.060**	0.97	0.94–0.99	0.033	0.98	0.95–1.01	**0.219**
CHEI	0.99	0.98–1.00	**0.193**	0.99	0.98–1.00	0.125	0.99	0.98–1.01	**0.388**
MD	1.02	0.93–1.12	**0.688**	0.98	0.89–1.09	0.719	1.01	0.91–1.12	**0.885**
Factor 1: Protein-rich	**0.76**	0.64–0.91	**0.002**	**0.81**	0.68–0.96	**0.015**	**0.82**	0.69–0.98	**0.033**
Factor 2: Vegetables	0.90	0.77–1.06	0.200	0.90	0.77–1.06	0.211	0.90	0.76–1.06	0.219
Factor 3: Sugar, oil and condiments	0.94	0.81–1.08	0.391	0.95	0.82–1.09	0.451	0.96	0.82–1.13	0.639
**555 subjects without Diabetes, Stroke, and Asthma**									
DASH	**0.95**	0.92–0.99	**0.013**	**0.95**	0.91–0.99	**0.007**	**0.96**	0.92–1.00	**0.032**
CHEI	0.99	0.98–1.00	0.136	0.99	0.97–1.00	0.115	0.99	0.98–1.01	0.272
MD	0.97	0.86–1.08	0.548	0.94	0.83–1.05	0.271	0.95	0.84–1.08	0.450
Factor 1: Protein-rich	**0.80**	0.66–0.97	**0.022**	**0.84**	0.69–1.00	**0.049**	0.84	0.68–1.03	0.085
Factor 2: Vegetables	0.86	0.70–1.06	0.148	0.87	0.71–1.06	0.176	0.84	0.67–1.06	0.139
Factor 3: Sugar, oil and condiments	0.89	0.75–1.06	0.182	0.90	0.75–1.08	0.248	0.91	0.74–1.12	0.389

CHEI: Chinese Healthy Eating Index; DASH: Dietary Approaches to Stop Hypertension; MD: Mediterranean Diet; OR: odds ratio; CI: 95% confidence interval. Note: Bold values denote statistical significance at the *p* value < 0.05 level. ^a^ Model 2 adjusted for sex and age. ^b^ Model 3 adjusted for sex, age, body mass index (BMI), energy intake, educational level, marital status, household income, smoking status, alcohol use, and doing housework.

## Supplementary Materials

The following are available online at https://www.mdpi.com/article/10.3390/ijerph182010852/s1. Table S1: Components and scoring of the CHEI, DASH, and MD. Table S2: Food groups and items in Food frequency questionnaire.

## Abbreviations

The following abbreviations are used in this manuscript:

DGC	Dietary Guidelines for Chinese;
SSAC	Shanghai Suburban Adult Cohort;
FFQ	food frequency questionnaire;
BMI	body mass index;
Cm	Centimeters;
Kg	Kilograms;
CES-D	Centers for Epidemiologic Studies Depression Scale;
CHEI	Chinese Healthy Eating Index;
DASH	Dietary Approaches to Stop Hypertension;
MD	Mediterranean Diet;
OR	odds ratio;
CI	95% confidence interval.
