# Supplementary material for: Association between Dietary Patterns and Frailty Prevalence in Shanghai Suburban Elders: A Cross-Sectional Study
Source: Int J Environ Res Public Health. 2021 Oct 15;18(20):10852. doi: 10.3390/ijerph182010852 (PMC8535511; doi:10.3390/ijerph182010852)
Supplement: Supplementary file 1 [file ijerph-18-10852-s001.zip › ijerph-1406922-supplementary.pdf]

Supplemental table S1. Components and scoring of the CHEI, DASH and MD

Component	Intake of minimum score	Intake of maximum score	Score range
CHEI-2016			0 ~ 100
1. Total Grains (sp)	0	≥ 2.5	0 ~ 5
2. Whole Grains and Mixed Beans (sp)	0	≥ 0.6	0 ~ 5
3. Tubers (sp)	0	≥ 0.3	0 ~ 5
4. Total Vegetables (sp)	0	≥ 1.9	0 ~ 5
5. Dark Vegetables (sp)	0	≥ 0.9	0 ~ 5
6. Fruits (sp)	0	≥ 1.1	0 ~ 10
7. Dairy (sp)	0	≥ 0.5	0 ~ 5
8. Soybeans (sp)	0	≥ 0.4	0 ~ 5
9. Nuts (sp)	0	≥ 0.4	0 ~ 5
10. Fish and Seafood (sp)	0	≥ 0.6	0 ~ 5
11. Poultry (sp)	0	≥ 0.3	0 ~ 5
12. Eggs (sp)	0	≥ 0.5	0 ~ 5
13. Red Meat (sp)	≥ 3.5	≤ 0.4	0 ~ 5
14. Cooking Oils (g)	≥ 32.6	≤ 15.6	0 ~ 10
15. Sodium (mg)	≥ 3608	≤ 1000	0 ~ 10
16. Added Sugars (% of Energy Intake)	≥ 20%	≤ 10%	0 ~ 5
17. Alcohol (g)	≥ 60 (male); ≥40(Female)	≤ 25(male); ≤15(Female)	0 ~ 5
DASH [a]			8 ~ 40
1. Fruits (sp)	Quintile 1	Quintile 5	1 ~ 5
2. Vegetables (sp)	Quintile 1	Quintile 5	1 ~ 5
3. Nuts and legumes (sp)	Quintile 1	Quintile 5	1 ~ 5
4. Low-fat dairy (sp)	Quintile 1	Quintile 5	1 ~ 5
5. Whole grains (sp)	Quintile 1	Quintile 5	1 ~ 5
6. Red and processed meats (sp)	Quintile 5	Quintile 1	1 ~ 5
7. Sugar-sweetened beverages (g)	Quintile 5	Quintile 1	1 ~ 5
8. Sodium (mg)	Quintile 5	Quintile 1	1 ~ 5
MD			0 ~ 9
1. Vegetables (sp)	Below the median	Above the median	0 ~ 1
2. Fruits (sp)	Below the median	Above the median	0 ~ 1
3. Nuts (sp)	Below the median	Above the median	0 ~ 1
4. Whole grains (sp)	Below the median	Above the median	0 ~ 1
5. Legumes (sp)	Below the median	Above the median	0 ~ 1
6. Fish and Seafood (sp)	Below the median	Above the median	0 ~ 1
7. Monounsaturated/saturated fat ratio	Below the median	Above the median	0 ~ 1
8. Red and processed meats (sp)	Below the median	Above the median	0 ~ 1
9. Alcohol (g)	<5, > 15	5 ~ 15	0 ~ 1

CHEI: Chinese Healthy Eating Index; DASH: Dietary Approaches to Stop Hypertension; MD: Mediterranean Diet; SP: Standard Portion; [a]: Intakes between the minimum and maximum levels are scored proportionately.

Supplemental table S2. Food groups and items in Food frequency questionnaire

Food groups	Items in Food frequency questionnaire
Rice and rice products	Rice dishes, congee, rice noodles and rice cakes, etc.
Wheat and wheat products	Noodles, bread, steamed stuffed bun and dumpling, etc.
Whole Grain and Mixed Beans	Sorghum, millet, oats, mung beans and red beans, etc.
Tubers	Sweet potato, yam, taro and potato, etc.
Light colored vegetables	Stem, melon, root, algae and mushroom, etc.
Dark vegetables	Dark-green leafy vegetables, red and yellow vegetables, etc.
Fruits	Apple, banana, orange, pear, grape or raisin, watermelon and strawberry, etc.
Juice	Pure fruit and vegetable juice.
Red meat	Pork, beef and lamb, etc.
Poultry	Chicken, duck and goose, etc.
Animal innards	Heart, liver, kidneys, lungs and blood, etc.
Ocean fish	Ribbonfish, croaker, salmon and pompano, etc.
Freshwater fish	Carp, silver carp, bass and carp, etc.
Shellfish, Shrimp and Crab	Clams, kiwi shrimp, crabs and sea crabs, etc.
Eggs	Eggs, duck eggs and quail eggs, etc.
Dairy and dairy products	fresh milk, yogurt, powdered milk and cheese, etc.
Nuts and Seeds	Peanuts, walnuts, almonds and melon seeds, etc.
Fried dough foods and potato chips	Fried dough sticks, doughnuts, instant noodles and potato chips, etc.
Processed meat	Bacon, sausage and luncheon meat, etc.
Sweets and desserts	Chocolate, candy, jelly, ice cream and pudding, etc.
Cakes, cookies, pies and biscuits	Cake, mooncake, snack and cracker, etc.
Soybean and soybean products	Soybean, bean curd, dried bean curd and bean curd bamboo, etc
Added sugars	Sugar, jam, and honey, etc.
Cooking oil	Oil, butter and margarine, etc.
Beverages	Carbonated beverage and sugar sweetened beverage, etc.
Alcoholic beverages	Chinese rice wine, beer, wine, white wine and whiskey, etc.
Condiments	Soy sauce, salad dressings, sauces, dips, ketchup, mustard and relish, etc.
